# The effect of ecological environmental changes and mollusciciding on snail intermediate host of *Schistosoma* in Qianjiang city of China from 1985 to 2015

**DOI:** 10.1186/s13071-020-04273-1

**Published:** 2020-08-05

**Authors:** Juan Qiu, Rendong Li, Hong Zhu, Jing Xia, Ying Xiao, Duan Huang, Yong Wang

**Affiliations:** 1 Key Laboratory of Monitoring and Estimate for Environment and Disaster of Hubei Province, Innovation Academy for Precision Measurement Science and Technology, Chinese Academy of Sciences, Wuhan, People’s Republic of China; 2 Hubei Center for Disease Control and Prevention, Hubei Provincial Academy of Preventive Medicine, Wuhan, People’s Republic of China; 3 Faculty of Geomatics, East China University of Technology, Nanchang, People’s Republic of China; 4 State Key Laboratory of Resources and Environmental Information Systems, Institute of Geographical Sciences and Natural Resources Research, Chinese Academy of Sciences, Beijing, People’s Republic of China

**Keywords:** Ecological environmental changes, Mollusciciding, *Schistosoma*, Snail control, Multilevel growth model

## Abstract

**Background:**

Schistosomiasis remains prevalent in Africa, Asia and South America with an estimated burden of 1.9 million disability-adjusted life years in 2016. Targeting snails as a key to success for schistosomiasis control has been widely approved, but the long-term quantitative effects of interventions on snail control that would inform and improve future control programmes are unclear. Over the last six decades, schistosomiasis in China had been brought largely under control, and snail control as supplementary methods or part of integrated multisectoral approaches in different historical periods has played an essential role.

**Methods:**

Ecological environment factors, prevalence and control data on *Oncomelania hupensis* between 1985 and 2015 at 5-year intervals in Qianjiang city, China, were collected. A multilevel growth model approach was used to examine the long-term effects of ecological environmental changes and mollusciciding on snail-infested area (SIA) and living snail density (LSD) during the 30 years.

**Results:**

The variation of SIA was 68.4% in spatial distribution, while the variation of LSD was 68.4% in temporal distribution. Continuous mollusciciding could result in significant LSD reduction, but may not lead to significant SIA reduction. The normalized difference vegetation index (NDVI), patch size coefficient of variation (PSCoV) and mean patch size (MPS) reduction, slightly due to eco-environmental changes decreased SIA, while mean perimeter-area ratio (MPAR) and dry farm-land proportion (DFLP) reduction might increase SIA. Only NDVI and MPAR reduction led to a lower LSD.

**Conclusions:**

Mollusciciding was more effective in reducing snail density, but it is not easy to eliminate snails completely. Environmental modifications could completely change the snail breeding environment and reduce its infestation area. Due to difficulty of scaling-up the current environmental modifications in waterway network regions, more effective snail control methods are needed. The experience in China could thereby provide guidance for other schistosomiasis endemic areas with a high snail prevalence.
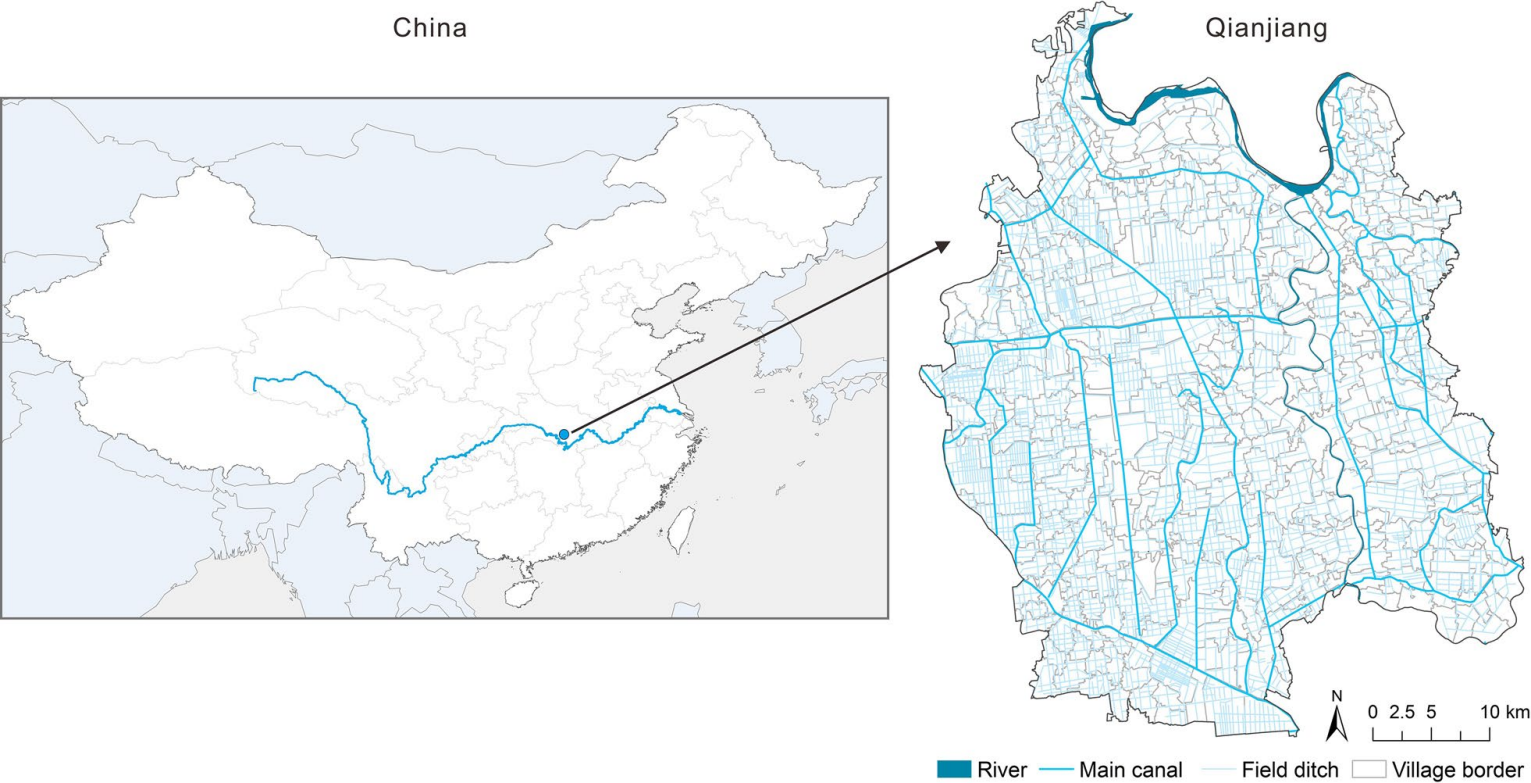

## Background

Schistosomiasis is a parasitic disease caused by trematode worms of the genus *Schistosoma*, and the burden of disease attributed to the parasite was estimated at 1.9 million disability-adjusted life years in 2016 [1–3]. Over the past decade, the global strategy for schistosomiasis has focused on the control of disease morbidity by scaling up targeted mass drug administration, also known as preventive chemotherapy [4, 5]. Recently, targeting snails (the intermediate hosts of *Schistosoma*, and living in specific natural environments) as a key to success for schistosomiasis control has been widely approved and should be included in national schistosomiasis control strategies for optimal disease control [6–9]. The WHO published new guidelines for field application methods for chemical-based snail control in 2017 [10], which underscores the renewed interest in the use of snail control. However, the long-term quantitative effects of interventions on snail control are unclear.

Although schistosomiasis is generally referred to as a neglected tropical disease, this is not the case in the People’s Republic of China [11]. During the last six decades, the control of this disease has been regarded as a top priority and the national control programmes had brought schistosomiasis japonica largely under control [12, 13]. *Oncomelania hupensis* (*O. hupensis*, the only intermediate host of *Schistosoma japonicum*) control as supplementary methods or part of integrated multisectoral approaches in different historical periods has played an essential role since 1956 [14]. Snail control measures include environmental modifications (agriculture, water conservancy and forestry projects) and/or mollusciciding [11, 15], called intentional ecological environmental interventions. We already know that snail-infested area and/or snail density, the two important epidemic indicators, were significantly reduced after the implementation of snail control measures [16, 17], but the spatiotemporal heterogeneity of the reduced trend and the differences in driving factors of snail-infested area and density reduction are not clear. In addition, the dynamics of snails is affected not only by snail control measures but also by unintentional ecological environmental interventions. The unintentional ecological environmental interventions refer to the natural or artificial non-targeted snail killing behaviors, including the growth and evolution of natural vegetation and the change of land use/cover due to the adjustments in the agricultural economic structure that may affect the breeding environment for snails. The ecological environmental impact on snail habitats has also been studied, and factors include temperature, humidity, vegetation and landscape pattern [18–22]. However, the combined effect of the environment and control measures on snails is unclear. To address these questions, we assessed the effect of all control measures and environmental factors on snail-infested areas and density using 30 years of data from Qianjiang city, China. The quantitative long-term experience will provide support for precise snail control.

Remote sensing provides the possibility to acquire surface domain data for long-term monitored ecological environments that affect snail survival. Combined with previous studies on snail survival factors [18–22], land use, humidity, vegetation, and landscape pattern from remote sensing will be discussed. Landscape pattern analysis can provide indications of whether an area offers a suitable habitat for snails, and the results of this type of analysis also provide recommendations for future landscape management activities with an aim to control snails [19, 21].

## Methods

### Parasitological data collection

**Fig. 1:**
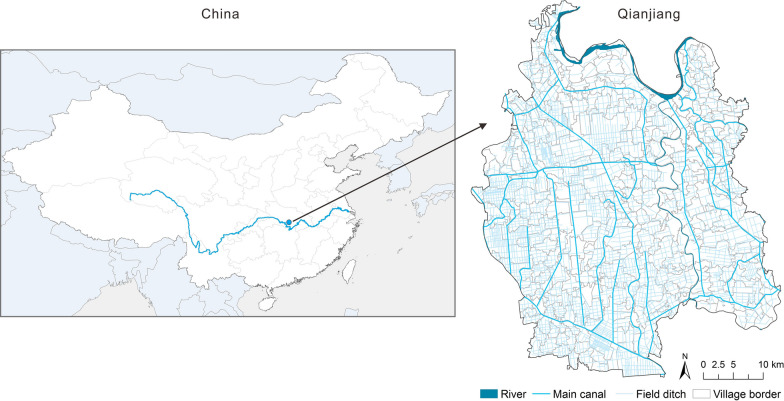
Qianjiang city location and the distribution of waterway networks. The waterway networks are extracted from the GaoFen 2 (GF-2) images (spatial resolution: 2.35 m) in 2016 which were downloaded from Hubei data and application network of the high-resolution earth observation system (http://hbeos.org.cn/), based on remote sensing image classification

The prevalence data on *O. hupensis* in Qianjiang city (Fig. 1) were obtained from the Data-Center of China Public Health Science (http://www.phsciencedata.cn) in 1985, 1990, 1995, 2000, 2005, 2010 and 2015. Data on snail-infested areas (SIA), living snail density (LSD) and mollusciciding (MOL, yes/no), were collected through village-based field repeated cross-sectional surveys conducted by health professionals from local schistosomiasis control stations according to the guide of the national schistosomiasis surveillance and the standard of prevention and control of schistosomiasis (http://www.chinacdc.cn). A total of 446 villages including 292 historical snail villages and 154 historical snail free villages were appended to the village-level administrative division coverage according to the name of the county, town, and village. The Yangtze River is located to the north of the study area. In 2014, the prevalence of *S. japonicum* infection in humans (positive blood test rate) was approximately 3.78%, and the snail-infested area was 1529.22 ha in this region [23]. The snail distribution was linear along the canals and ditches.

### Land-use classification

The 1:100,000-scale land-use data between 1985 and 2015 at 5-year intervals were provided by the Data Center for Resources and Environmental Sciences, Chinese Academy of Sciences (http://www.resdc.cn) using visual interpretation based on professional knowledge. The main classes were water bodies (lakes, rivers and ponds), dry or paddy agricultural land, forestland, water channels, and construction land. Dry farmland proportion (DFLP) and paddy farmland proportion (PFLP) in villages were calculated after the land-use data were intersected with village-level administrative division coverage.

### Landscape pattern

A village was considered the scale of the study of the landscape patterns of *O. hupensis*. The village boundary coverage in the study area was overlaid on the land-use coverage whose features (elements) represented landscape patches and landscape metrics of each patch at the patch level. Each village at the landscape level was calculated using ArcGIS 10.3 (ESRI Inc., Redlands, CA, USA) and Patch Analyst version 5 (http://www.cnfer.on.ca/SEP/patchanalyst/). The software recognizes more than 20 landscape metrics [24]. Based on their ecological significance and to avoid information redundancy, we selected the area metrics, edge metrics, patch density, patch size, variability metrics and shape metrics listed in Table 1.

**Table 1:** Summary of independent variables used in the analysis of SIA and LSD, with abbreviation and unit

Category	Variable	Abbreviation	Units
Land use	Dry farmland proportion	DFLP	%
	Paddy farmland proportion	PFLP	%
Landscape pattern	Area metrics		
	Class area	CA	ha
	Edge metrics		
	Total edge	TE	m
	Edge density	ED	m/ha
	Mean patch edge	MPE	m/#
	Patch density, patch size and variability metrics		
	Number of patches	NumP	#
	Mean patch size	MPS	ha
	Median patch size	MedPS	ha
	Patch size standard deviation	PSSD	ha
	Patch size coefficient of variation	PSCoV	%
	Shape metrics		
	Mean shape index	MSI	
	Mean perimeter-area ratio	MPAR	
	Mean patch fractal dimension	MPFD	
	Area-weighted mean patch fractal dimension	AWMPFD	
	Area-weighted mean shape index	AWMSI	
Remote sensing environmental indicators	Normalized difference vegetation index	NDVI	
	Wetness index	WI	
Distance	Shortest distance from the Yangtze river	SDYR	km
Snail control measure	Whether mollusciciding had taken place	MOL (Y/N)	

### Remote sensing environmental indicators

The normalized difference vegetation index (NDVI) and wetness index (WI) were extracted from Landsat images through band operation (the ratio of the difference between the near-infrared band and the visible band and the sum of the two bands) and tasseled cap transformation [25] using ERDAS IMAGINE 2013 software (ERDAS Inc., Atlanta, GA, USA). The images covering the study area with a spatial resolution of 30 m acquired in July 1987, July 1992, July 1995, July 2000, August 2006, July 2010 and July 2015 were downloaded from the U.S. Geological Survey (USGS; http://glovis.usgs.gov/).

### The shortest distance from the Yangtze River (SDYR)

The shortest distance of each village from the Yangtze River was calculated using ArcGIS 10.3 (ESRI Inc., Redlands, CA, USA).

### Statistics

The long-time series snail survey data belong to longitudinal repeated measurement data with hierarchical structure and group effect (between-group heterogeneity and within-group homogeneity). In order to exclude intra-class correlation, a multilevel modeling approach was adopted. We followed the methods described by Singer & Willett [26] and fitted a taxonomy of multilevel longitudinal growth models to partition the variance into mean SIA or LSD within and between villages and to contrast the effects of ecological environment and mollusciciding between SIA and LSD.

First, an unconditional means model (Model A) was fitted to provide a baseline for future model comparisons, which were used to the calculate an intraclass correlation coefficient (ICC) to measure both between-group heterogeneity and within-group homogeneity [27]. The unconditional means model can be viewed as a two-level model as follows:

$$ {\text{Level-1 model }}\left( {\text{within-village}} \right):Y_{ij} = \beta_{0i} + \varepsilon_{ij} $$

where $Y_{ij}$ is the village $i$’s SIA or LSD on occasion $j$, $\beta_{0i}$ is the mean outcome for the *i*th village, $\varepsilon_{ij  }$ is the level-1 error, where $\varepsilon_{ij  }$ ∼ N (0, $\sigma_{\varepsilon }^{2}$), and represents the within-village variability.

$$ {\text{Level-2 model }}\left( {\text{village-level}} \right):\beta_{0i} = \gamma_{00} + {\mathcal{U}}_{0i} $$

where $\gamma_{00}$ represents the overall mean SIA or LSD across all measurement occasions and villages, ${\mathcal{U}}_{0i}$ represents the degree to which there is variation in *Y* between villages, and ${\mathcal{U}}_{0i}$ ∼ N (0, $\sigma_{0}^{2}$), and $\sigma_{0}^{2}$ represents the between-village variability in *Y*. The ICC can then be calculated as follows: ICC = $\widehat{\sigma }_{0}^{2} /\left( {  \widehat{\sigma }_{0}^{2} + \widehat{\sigma }_{\varepsilon }^{2} } \right)$. The $\sigma_{0  }^{2}$ and $\sigma_{\varepsilon }^{2}$ differed from zero, in addition to the statistically significant variance will justify the use of the multilevel models [28].

Secondly, an unconditional growth model (Model B) was formulated as a linear function of a time variable plus random level-1 error to specify a level-1 model that can best describe the within-village SIA or LSD growth over time. The time variable, TIME, was set to zero for the initial measurement occasion, 1985, so that the level-1 intercept, $\gamma_{00} + {\mathcal{U}}_{0i}$, represented the expected SIA or LSD value for village $i$ in 1985. For each subsequent 5 years, 1 was added until 2015. The equations for the unconditional growth model were specified as follows:

$$ {\text{Level-}}1:\, Y_{ij} = \beta_{0i} + \beta_{1i} {\text{TIME}}_{ij} + \varepsilon_{ij} $$

$$ {\text{Level-}}2:\, \beta_{0i} = \gamma_{00} + {\mathcal{U}}_{0i} ;\beta_{1i} = \gamma_{10} + {\mathcal{U}}_{1i} $$

where $\gamma_{10}$ is the average 5-year rate of change in *Y* across all villages, ${\mathcal{U}}_{1i}$ referred to as a random effect of TIME. The level-1 and level-2 error terms ($\varepsilon_{ij}$, ${\mathcal{U}}_{0i}$, ${\mathcal{U}}_{1i}$) are assumed to be normally distributed, and the variances of these error terms ($\sigma_{\varepsilon }^{2}$, $\sigma_{0}^{2}$, $\sigma_{1}^{2}$) are homoscedastic. The covariance of ${\mathcal{U}}_{0i}$ and ${\mathcal{U}}_{1i}$ is $\sigma_{01}^{2}$. The assumption of normality is not satisfied through a Quantile-Quantile (Q-Q) plot test of the quantiles of the residuals against the quantiles of a specified theoretical distribution [29]. With this violation of the normality assumption, and to avoid parameter estimates biased, REML (restricted estimation maximum likelihood) was used [29].

As a third step, to examine the significant time-varying covariates, level-1 covariates $X_{qij}$ (Table 1) were then added as a fixed effect to the unconditional growth model (Model C) after assessing collinearity. Variables with relatively high collinearity were excluded, as assessed by examining the correlation coefficient and the variance inflation factors (VIFs).

$$ {\text{Level-}}1:\,Y_{ij} = \beta_{0i} + \beta_{1i} {\text{TIME}}_{ij} + \mathop \sum \limits_{q = 1}^{Q} \beta_{q} X_{qij} + \varepsilon_{ij} $$

$$ {\text{Level-}}2:\,\beta_{0i} = \gamma_{00} + {\mathcal{U}}_{0i} ;\beta_{1i} = \gamma_{10} + {\mathcal{U}}_{1i} $$

where Q is the number of level-1 covariates.

Finally, non-correlated level-1 covariates (α-level > 0.05) were excluded, and SDYR was added to model C to control the village background covariate in the growth model (Model D).

$$ {\text{Level-}}1:\,Y_{ij} = \beta_{0i} + \beta_{1i} {\text{TIME}}_{ij} + \mathop \sum \limits_{q = 1}^{Q} \beta_{q} X_{qij} + \varepsilon_{ij} $$

$$ {\text{Level-}}2:\,\beta_{0i} = \gamma_{00} + \gamma_{01} {\text{SDYR}}_{i} + {\mathcal{U}}_{0i} ;\beta_{1i} = \gamma_{10} + \gamma_{11} {\text{SDYR}}_{i} + {\mathcal{U}}_{1i} $$

The combined model: $Y_{ij} = \gamma_{00} + \gamma_{01} {\text{SDYR}}_{i} + \gamma_{10} {\text{TIME}}_{ij} + \gamma_{11} {\text{SDYR}}_{i} *{\text{TIME}}_{ij} + \mathop \sum \limits_{q = 1}^{Q} \beta_{q} X_{qij} + {\mathcal{U}}_{0i} + {\mathcal{U}}_{1i} {\text{TIME}}_{ij} + \varepsilon_{ij}$

All analyses were conducted using SAS version 9.3 MIXED procedure (SAS Institute, Inc., Cary, NC, USA).

## Results

### Model A

Estimates of the variance parameters in the SIA and LSD Model A for the village-specific residual ($\widehat{\sigma }_{\varepsilon }^{2}$ = 31.195 and 0.459, respectively) and mean SIA and LSD ($\widehat{\sigma }_{0}^{2}$ = 67.561 and 0.214, respectively) both differed from zero, and these values in addition to the statistically significant variance justified the use of the multilevel models. The ICC calculated as 67.561/(67.561 + 31.195) = 0.684 for SIA and 0.214/(0.214 + 0.459) = 0.318 for LSD, respectively, indicated that approximately 68.4% of the variation in SIA and 31.8% of the variation in LSD are attributable to differences between villages.

### Model B

This model resulted in statistically significant fit improvement with reduced AIC and BIC (Table 2). The linear fixed effect of TIME differed significantly and negatively from zero both in the SIA and LSD models, indicating that villages’ SIA and LSD significantly decline every 5 years. The estimated covariance $\widehat{\sigma }_{01}^{2}$ = − 0.337 and − 25.677 in the SIA and LSD models, respectively, suggested a negative correlation between initial status and rates of change in SIA and LSD, which indicated that villages with initially high SIA or LSD declined relatively quickly compared to villages with lower SIA or LSD.

**Table 2:** Parameter estimates, standard errors and fit statistics for snail-infested area and living snail density model

Variables	Snail-infested area (SIA)				Living snail density (LSD)			
	Model A	Model B	Model C	Model D	Model A	Model B	Model C	Model D
Fixed effects								
Intercept, $\gamma_{00}$	4.423 ± 0.405***	6.816 ± 0.693***	15.962 ± 13.016	13.428 ± 2.840***	0.288 ± 0.025***	0.647 ± 0.068***	0.913 ± 0.746	2.007 ± 0.260***
TIME, $\gamma_{10}$		− 0.676 ± 0.100***	− 0.591 ± 0.108***	− 2.292 ± 0.424***		− 0.105 ± 0.013***	− 0.068 ± 0.013***	− 0.403 ± 0.050***
MOL (0/1), $\beta_{1}$			3.811 ± 0.646***	3.142 ± 0.667***			0.691 ± 0.078***	0.542 ± 0.081***
DFLP, $\beta_{2}$			− 0.027 ± 0.010**	− 0.020 ± 0.010*			− 0.001 ± 0.001	
PSCoV, $\beta_{3}$			0.016 ± 0.003***	0.014 ± 0.003***			0.0004 ± 0.0002	
MPFD * 100,$\beta_{4}$			− 0.034 ± 0.051				− 0.003 ± 0.003	
MPS, $\beta_{5}$			0.034 ± 0.013*	0.035 ± 0.013**			0.0003 ± 0.0007	
MPAR * 0.001, $\beta_{6}$			− 0.012 ± 0.005*	− 0.012 ± 0.005*			0.004 ± 0.001***	0.003 ± 0.001***
AWMPFD * 100, $\beta_{7}$			− 0.080 ± 0.086				− 0.002 ± 0.005	
NDVI, $\beta_{8}$			4.882 ± 0.623***	4.863 ± 0.622***			0.306 ± 0.070***	0.291 ± 0.068***
WI, $\beta_{9}$			− 0.020 ± 0.018				− 0.002 ± 0.002	
MOL × TIME, $\beta_{10}$			− 0.285 ± 0.126*	− 0.175 ± 0.133			− 0.113 ± 0.016***	− 0.079 ± 0.016***
SDYR (km, $\gamma_{01}$				− 0.272 ± 0.057***				− 0.036 ± 0.005***
SDYR × TIME, $\gamma_{11}$				0.037 ± 0.009***				0.007 ± 0.001***
Random effects								
Level 1 (residual)								
Within village, $\sigma_{\varepsilon }^{2}$	31.195 ± 0.910***	13.118 ± 0.420***	12.307 ± 0.396***	12.316 ± 0.395***	0.459 ± 0.013***	0.190 ± 0.006***	0.186 ± 0.006***	0.187 ± 0.006***
Level 2								
Initial status, $\sigma_{0}^{2}$	67.561 ± 4.855***	198.160 ± 14.139***	178.850 ± 12.948***	170.480 ± 12.278***	0.214 ± 0.019***	1.836 ± 0.136***	1.475 ± 0.115***	1.338 ± 0.105***
Rate of change, $\sigma_{1}^{2}$		3.616 ± 0.287***	3.561 ± 0.285***	3.393 ± 0.271***		0.061 ± 0.005***	0.051 ± 0.004***	0.045 ± 0.004***
Rate of change covariance, $\sigma_{01}^{2}$		− 25.677 ± 1.955***	− 24.246 ± 1.869***	− 23.075 ± 1.774***		− 0.337 ± 0.025***	− 0.275 ± 0.022***	− 0.248 ± 0.020***
Fit statistics								
AIC	18723.900	17058.800	16893.800	16873.800	6455.000	4486.600	4428.600	4343.900
BIC	18732.100	17075.100	16910.200	16890.200	6463.200	4503.000	4445.000	4360.300

*P ≤ 0.05, **P ≤ 0.01, ***P ≤ 0.001

### Model C

PFLP, CA, TE, ED, MPE, NumP, MedPS, PSSD, MSI and AWMSI were excluded after assessing collinearity, and Model C added fixed effects for the remaining 10 significant independent variables (Table 1) to Model B. The results from Model C (Table 2) indicated that DFLP, PSCoV, MPS, MPAR, and NDVI were statistically significant predictors of SIA. However, only MPAR and NDVI were statistically significant predictors of LSD.

### Model D

The results from the final Model D (Table 2) indicated that even after adjusting for the differences in SDYR and the interaction between SDYR and TIME, the environmental predictors that appeared in Model C, such as DFLP, PSCoV, MPS, MPAR, and NDVI, remained statistically significant in Model D for SIA, as did MPAR and NDVI for LSD. A lower DFLP ($\widehat{\beta }_{2}$ = − 0.02, *P* = 0.036) and MPAR ($\widehat{\beta }_{6}$ = − 0.012, *P* = 0.02) was associated with a higher SIA, but a higher PSCoV ($\widehat{\beta }_{3}$ = 0.014, *P* < 0.001), MPS ($\widehat{\beta }_{5}$ = 0.035, *P* = 0.006) and NDVI ($\widehat{\beta }_{8}$ = 4.863, *P* < 0.001) were associated with a higher SIA. At the same time, a higher MPAR ($\widehat{\beta }_{6}$ = 0.003, *P* < 0.001) and NDVI ($\widehat{\beta }_{8}$ = 0.291, *P* < 0.001) were associated with a higher LSD. The linear fixed effects of time ($\gamma_{10}$) in Model D were smaller than those in Models B or C, indicating that the SIA and LSD decreased more quickly after controlling for village background and time-varying covariates. Villages with chemical mollusciciding had higher SIA and LSD compared to that of villages with no mollusciciding ($\widehat{\beta }_{1}$ = 3.142 and 0.542, *P* < 0.001 and *P* < 0.001, respectively). The interaction effect among TIME and MOL that appeared in Model C remained statistically significant in Model D for LSD ($\widehat{\beta }_{10}$ = − 0.079, *P* < 0.001); however, that value was no longer statistically significant for SIA ($\widehat{\beta }_{10}$ = − 0.175, *P* = 0.190). A lower SDYR was associated with a higher SIA and LSD ($\widehat{\gamma }_{01}$ = − 0.272 and − 0.036, *P* < 0.001, and *P* < 0.001, respectively). Furthermore, the interaction of SDYR and TIME was statistically significantly positive in Model D for both SIA and LSD ($\widehat{\gamma }_{11}$ = 0.037 and 0.007, *P* < 0.001, and *P* < 0.001, respectively). Similar to Models B and C, the random effects ($\widehat{\sigma }_{\varepsilon }^{2}$, $\widehat{\sigma }_{0}^{2}$, $\widehat{\sigma }_{1}^{2}$, and $\widehat{\sigma }_{01}^{2}$) remained statistically significant in Model D, and all of which were lowest in Model D for both SIA and LSD. Among the models, Model D had the smallest AIC and BIC for both SIA and LSD.

### Temporal trend of factors

**Fig. 2:**
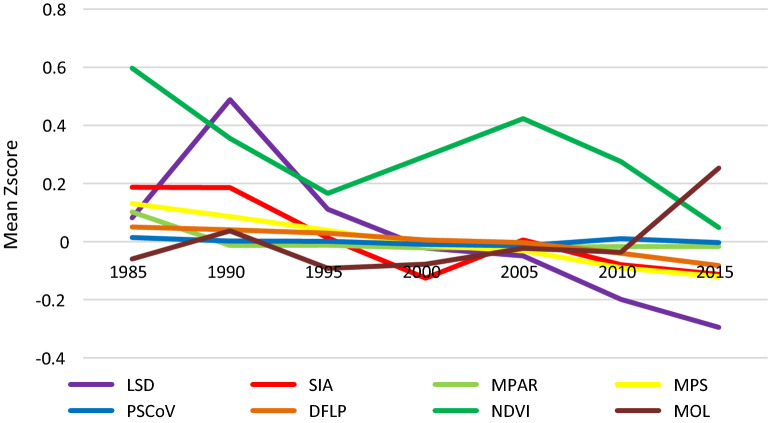
Annual mean Z-score of significant time-varying covariates and dependent variables

All significant time-varying covariates and dependent variables were Z-score standardized to show their temporal trend. Overall, the annual mean Z-scores of LSD, SIA, MPAR, MPS, PSCov, DFLP and NDVI decreased slightly, while the annual mean of MOL increased between 1995 and 2015 (Fig. 2).

## Discussion

### The multilevel modeling approach

The ICC value, random effect and their significance, as well as the model fitting statistics (AIC and BIC) which reduced from Model A to Model D, justified the use of the multilevel models. By using the multilevel modeling approach to analyze the snail longitudinal data, not only the heterogeneity of the spatial and temporal distribution of SIA and LSD can be revealed, but also the influencing factors and their interactions can be analyzed.

### Spatiotemporal heterogeneity

The spatial and temporal variation characteristics of SIA and LSD were different. The variation in SIA was 68.4% (ICC = 68.4%) in space, and the variation in LSD was 68.2% (ICC = 31.8%, 1–31.8% = 68.2%) in time. That is, the SIA has an obvious difference in its spatial distribution, while the LSD has an obvious change in its temporal distribution. The distribution area of snails in China has been maintained at 3.5–3.8 billion m^2^ in the past 10 years (2010–2017) [30], which also shows that the interannual variation in the SIA is not significant. The significant heterogeneity of the SIA’s spatial distribution suggests the importance of identifying its spatial pattern and locating its hot spots.

### Landscape pattern

In lake and marshland regions, snails present a planar distribution, with a large distribution range but a low density, but in plain regions with waterway networks (including irrigation canals and ditches), snails present a linear distribution, with a small distribution area but a relatively high density [31, 32]. Moreover, the density of snails in large ditches (width 1–3 m) might be lower than that in small ditches (width < 1 m) [33]. Our research confirms the above viewpoints from the perspective of landscape ecology. The MPAR indicates the complexity of patch shape, and the more complex or spindly the patch shape is, the larger the MPAR. The MPARs of river or lake beaches were smaller than that of ditches, and the MPARs of large ditches were smaller than that of small ditches, thus high MPARs led to a low SIA but a high LSD. The MPS increases as the patch size increases. Thus, river or lake beaches provide more suitable snail habitats than ditches. On the other hand, the SIA was positively related to PSCoV, which is used to measure the degree of landscape discretization. A decreased PSCoV indicates less intense and frequent human activity [34]. Thousands of waterway networks in Qianjiang were due to farmland reclamation from lakes and large-scale excavation channels after the mid-1970s, and 57.95% of the total number of channels had snails [31], and after 1990, various drainage and irrigation channels were finalized and human transformation activities reduced [35]. This may be the reason for the decrease of PScov and subsequently SIA.

### Vegetation and wetness

The effect of NDVI was different at different spatial scales as well as in different geomorphic type areas [19, 21]. At the village level in plain regions with waterway networks, our study found that a high NDVI indicated that relatively high vegetation cover led to a high SIA and LSD. The wetness showed no statistically significant association with SIA and LSD. This result was possibly attributed to the dominant cultivated land-use type and the same elevation in the plain water network area, which resulted in no regional difference in humidity at the village level.

### Other environmental factors

We found that SIA was negatively associated with DFLP, which confirmed the effectiveness of conversion of paddy fields into dry crop farming to eliminate snails [15]. DFLP, PSCoV and MPS were significantly correlated with SIA but not with LSD, indicating that the change in land use and patch density, patch size or variability pattern had a significant effect on the SIA but had no significant effect on LSD. The distance from the Yangtze River would affect the underground water level. The closer to the Yangtze River, the higher the humidity is in the surface soil, making it more suitable for snails, but this area had more SIA and LSD reduction over time.

### Mollusciciding

The snail environmental modification projects only involved large rivers with small coverage areas [36]. However, snails are mainly distributed along channels, including main canals, branch canals, lateral canals, agriculture ditches, and sublateral canals, which are classified according to size and purpose [19, 36]. Therefore, snail control mainly relies on chemical mollusciciding. In the process of eliminating snails, where there are snails, there are chemical molluscicides, and where the SIA or LSD is higher, there is a greater possibility of chemical mollusciciding. Therefore, mollusciciding becomes a symptom of the severity of the snail epidemic. The negative interaction effect among TIME and MOL indicated that the cumulative time effect of mollusciciding on LSD reduction was significant, but the effect on SIA reduction was not significant.

### Temporal dynamics of factors

The NDVI reduction might due to the cement lining of irrigation ditches, and MPS or PSCoV reduction through farmland reclamation and cultivation would reduce suitable snail habitats, while MPAR reduction caused by farmland reclamation and cultivation would increase them. Unintentional interventions led to ecological environmental changes, such as cultivated land consolidation, also reduced MPAR, MPS and PSCoV. The DFLP reduction probably occurred because of large-scale crawfish and rice co-cropping to increase agricultural economic effects. However, the possible complex effect of farmed crawfish on snail conditions remains to be further studied [35].

Under the combination of mollusciciding and ecological environmental changes caused by environmental modification, the SIA and LSD were significantly reduced, and the location with the originally higher SIA and LSD experienced a greater reduction. Continuous mollusciciding could result in significant LSD reduction, but not in significant SIA reduction. Environmental factors have changed only slightly over the past 30 years, and the natural environment suitable for snail breeding still largely exists. Thus, the question remains as to what happens when LSD decreases to a certain level? In fact, in recent years (2016–2018), snail has been in a low-prevalence state in large areas of China, and this low-prevalence state will remain for a long time [13, 30]. Furthermore, the risk of recurrence still remains due to environmental and sociopolitical changes [37]. The process for improving the efficiency of snail control under a low-prevalence states needs further study and would be a great challenge. Digging new and filling old small irrigation canals or even replacing ditch irrigation with drip irrigation would be an option to completely change the snail breeding environment.

## Limitations

Snail control methods which cannot be detected by environmental indicators, such as burying soil with sands and burning snails, were ignored. However, these methods are not universal and rarely involved. In addition, we could not distinguish between unintentional interventions and intentional environmental modifications on snail control. Finally, we did not assess factors that contribute to the snail spread, such as flooding. It is hoped that these questions would be addressed in future studies.

## Conclusions

Mollusciciding would be more effective in reducing snail density, but it is not easy to eliminate snails completely. Environmental modifications could completely change the snail breeding environment and reduce its infestation area. Due to the difficulty of scaling-up the current environmental modifications in waterway network regions, more effective snail control methods are needed. Our intervention research on schistosome-hosting snails could help in the development of snail control plans and selection of control methods, especially in sub-Saharan Africa with a high prevalence of snail intensity but few concrete snail control measures, which would further promote the goal to eliminate schistosomiasis.

## Data Availability

Data supporting the conclusions of this article are included within the article. The datasets used and/or analysed during the present study are available from the corresponding author on reasonable request.

## Abbreviations

SIA: snail-infested area
LSD: living snail density
NDVI: normalized difference vegetation index
PSCoV: patch size coefficient of variation
MPS: mean patch size
MPAR: mean perimeter-area ratio
DFLP: dry farm-land proportion
PFLP: paddy farmland proportion
WI: wetness index
MOL: mollusciciding
ICC: intraclass correlation coefficient
